# Dietary Flavonoids: Mitigating Air Pollution’s Cardiovascular Risks

**DOI:** 10.3390/nu16162647

**Published:** 2024-08-10

**Authors:** Oscar Andrés Rocha-Velasco, María Morales-Suárez-Varela, Agustín Llopis-González

**Affiliations:** 1 Research Group in Social and Nutritional Epidemiology, Pharmacoepidemiology and Public Health, Department of Preventive Medicine and Public Health, Food Sciences, Toxicology and Forensic Medicine, Faculty of Pharmacy and Food Sciences, Universitat de València, Av. Vicent Andrés Estelles s/n, 46100 Burjassot, Spain; osrove@alumni.uv.es (O.A.R.-V.); agustin.llopis@uv.es (A.L.-G.); 2 Biomedical Research Center in Epidemiology and Public Health Network (CIBERESP), Carlos III Health Institute, Av. Monforte de Lemos 3-5 Pabellón 11 Planta 0, 28029 Madrid, Spain

**Keywords:** dietary flavonoids, cardiovascular diseases, air pollution, oxidative stress, anti-inflammatory, public health

## Abstract

Air pollution significantly impacts cardiovascular health, yet pollution reduction strategies in cardiovascular disease prevention remain limited. Dietary flavonoids show promise in protecting cardiovascular health, but their potential to mitigate air-pollution-induced risks is unexplored. This study investigates this research gap. Following PRISMA-ScR guidelines, literature from 2014–2024 was searched across MedLine/PubMed, ScienceDirect, and MDPI databases. Of 463 identified studies, 53 were eligible for analysis based on PICO criteria. Findings revealed significant impacts of air pollution on cardiovascular health, including increased disease risks and mortality. Flavonoid intake demonstrated protective effects against these risks. Flavonoid mechanisms include improved endothelial function, antioxidant and anti-inflammatory effects, blood pressure regulation, antiplatelet effects, cardioprotection, and enhanced lipid and glucose metabolism. Higher flavonoid intake was consistently associated with reduced cardiovascular risks. While reducing pollution remains crucial, promoting flavonoid-rich diets is a promising complementary strategy. Public health initiatives should raise awareness about these benefits. Further research on direct interactions between flavonoid intake and air pollution exposure is needed. Current evidence supports integrating dietary interventions into broader strategies to reduce air pollution’s cardiovascular impacts.

## 1. Introduction

Environmental pollution, particularly air pollution, has emerged as the largest cause of premature reversible death and disability worldwide, posing a significant global health concern with substantial impacts on cardiovascular health [1]. In 2016 alone, ambient fine particulate matter (PM2.5) air pollution was responsible for an estimated 4.2 million deaths annually [2]. Air pollution is a complex mixture of gaseous and particulate components, including nitrogen dioxide (NO_2_), sulfur dioxide (SO_2_), ozone (O_3_), carbon monoxide (CO), and particulate matter of various sizes [3,4,5]. Fine (PM2.5) and ultrafine (PM0.1) particulates, primarily produced through fossil fuel combustion, pose significant health risks due to their ability to penetrate deep into the lungs and enter the bloodstream [4,6].

The cardiovascular risks associated with air pollution are extensive and well documented and include ischemic heart disease, stroke, hypertension, atrial fibrillation, and heart failure [7,8]. Both short-term and long-term exposure to air pollutants have been linked to increased risks of acute cardiovascular events and the development of chronic cardiovascular conditions [9,10,11,12,13,14]. The mechanisms underlying these effects are multifaceted and involve oxidative stress [15,16,17], systemic inflammation [18,19], endothelial dysfunction [18,20], and alterations in blood pressure regulation [21,22,23]. Importantly, even at concentrations below current regulatory standards, air pollution has been associated with increased cardiovascular risks [6,24].

The burden of disease attributable to air pollution is substantial, with significant economic costs to health and the environment [25,26]. Vulnerable groups, such as children, elderly individuals, individuals with preexisting conditions, and individuals with lower socioeconomic status, face greater risks from the cardiovascular effects of air pollution [27]. Despite its significant impact, pollution reduction has received little attention in cardiovascular disease (CVD) control programs and prevention guidelines [1]. The incorporation of pollution reduction strategies for cardiovascular disease prevention could save millions of lives, highlighting the urgent need for comprehensive approaches to address this critical public health challenge.

Given these challenges, nutrition is crucial for maintaining cardiovascular health and reducing major CVD risks [28]. Flavonoids have emerged as promising compounds in this context. Also known as bioflavonoids, flavonoids are a diverse group of polyphenolic secondary metabolites found in many fruits, vegetables, tea, cocoa, wine, nuts, seeds, spices, and other plant-based foods [29,30,31,32,33,34,35,36]. Flavonoids are synthesized by plants as part of their defense mechanisms and contribute to the color, flavor, and nutritional value of many fruits and vegetables. Several reviews have examined their food sources and the bioavailability, metabolism, and biological activity of these compounds in humans.

Flavonoids share a common basic structure but differ in the number and arrangement of hydroxyl groups, as well as other substituents. Chemically, flavonoids have the general structure of a 15-carbon skeleton arranged in a C6–C3–C6 configuration, which consists of two phenyl rings (A and B) and a heterocyclic ring (C) containing oxygen [37]. Flavonoids are classified into different families based on the degree of oxidation and pattern of substitution of the C-ring and the position of the B-ring. These chemical structures and variations allow flavonoids to exhibit a wide range of biological activities, making them important compounds in nutrition, medicine, and plant science.

**Figure 1:**
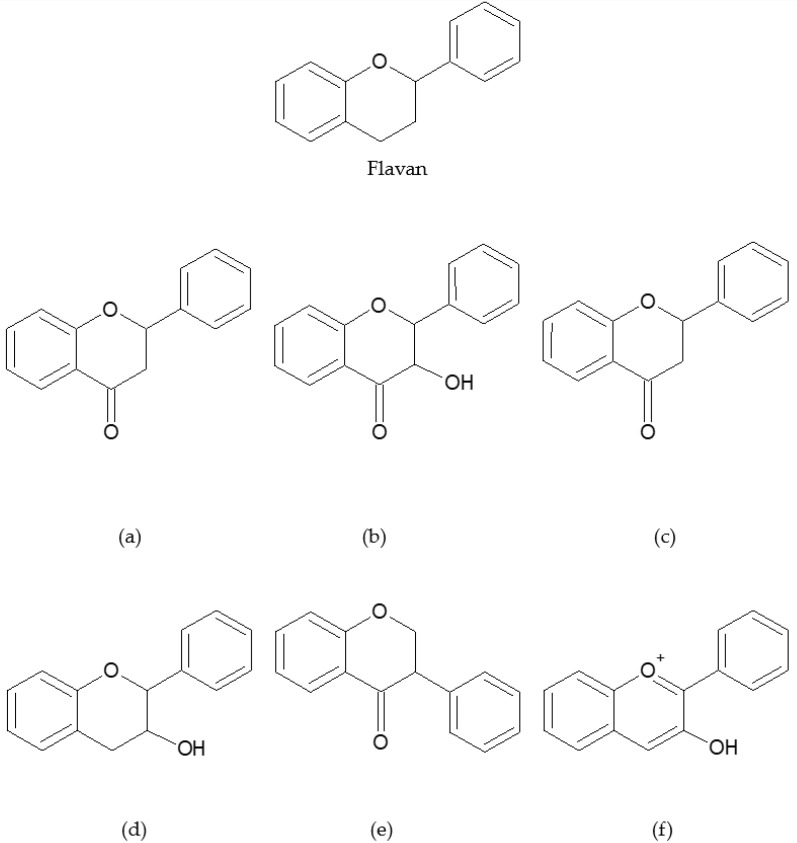
Chemical structures of flavonoids. (**a**) Flavones (**b**) Flavonols (**c**) Flavanones (**d**) Flavanols (**e**) Isoflavones (**f**) Anthocyanidins.

Flavonoids fall into six distinct classes based on their chemical structure (Figure 1): flavones (the B-ring is attached to the C2 position of the C-ring, with a double bond between C2 and C3 and a ketone at C4), flavonols (similar to flavones but with a hydroxyl group at the C3 position), flavanones (the B-ring is attached to the C2 position of the C-ring, with a single bond between C2 and C3 and a ketone at C4), flavanols (similar to flavanones but with a hydroxyl group at the C3 position and no ketone group at C4), isoflavones (the B-ring is attached to the C3 position of the C-ring), and anthocyanidins (similar to flavonols but without a ketone at C4 and typically with a positive charge on the oxygen in the C-ring) [34,35,38,39,40].

A healthy lifestyle, including a diet rich in these plant-based foods, is associated with the prevention of inflammatory diseases and improved cardiovascular health [41,42]. Flavonoids have gained attention for their potential cardiovascular-protective benefits, as indicated by epidemiological studies from the 1990s and 2000s [28]. They modulate genes related to metabolism, stress defense, and detoxification, and their molecular actions include antioxidant effects and the modulation of key enzymatic pathways [35,42].

The beneficial properties of flavonoids, such as their antioxidative, anti-inflammatory, antimutagenic, and anticarcinogenic effects, have been well documented [34,43]. These compounds have shown promise in protecting against CVDs, as supported by *in vitro* and *in vivo* studies [28,36].

There are few studies centered on the mean flavonoid ingestion [44,45,46,47]. A Spanish study found a median and mean of total flavonoids of 269.17 and 313.26 mg/day, respectively [46]. A wider European study found a mean intake of total flavonoids of 428 ± 49 mg/day, of which 136 ± 14 mg/day was of monomeric compounds and with the lowest intakes observed in Mediterranean countries [47]. A study on Australian adults found total flavonoid intake was 626 ± 579 mg/day [44] while a study on American adults found mean total flavonoid intake was 219 mg/day [45].

Human intervention studies have explored the administration of flavonoids through whole foods, dietary supplements, or individual compounds, highlighting their role in preserving cardiovascular health and reducing the risk of major CVDs [28,38,43]. Therefore, incorporating different types of flavonoids into the daily diet is highly recommended and justifiable for mitigating the risk of life-threatening diseases, such as CVD [35].

The current literature does not directly address the association between dietary flavonoid intake and the mitigation of cardiovascular health risks caused by air pollution. Through a comprehensive review of the literature published between 2014 and 2024, we summarize the existing evidence on the association between air pollution and CVD risk, examine the cardiovascular benefits of dietary flavonoids, and hypothesize that these two factors may interact. Our goal is to explore this interaction as a complementary strategy within a broader public health approach to mitigate the impact of air pollution on cardiovascular health. This research seeks to bridge the gap in current understanding and provide insights for future studies and potential interventions.

## 2. Methods

This narrative review was conducted following the Preferred Reporting Items for Systematic Reviews and Meta-Analyses extension for Scoping Reviews guidelines [48].

A comprehensive literature search was performed up to 19 June 2024 across the MedLine/PubMed, ScienceDirect, and MDPI databases. The search focused on the following terms: (air pollution OR air pollutants OR environmental pollutants OR particulate matter OR smog OR toxic air OR ambient air pollution) AND (dietary flavonoids OR flavonoid-rich foods OR antioxidants in diet OR polyphenols OR plant-based antioxidants OR quercetin OR catechins OR flavonols OR anthocyanins) AND (cardiovascular diseases OR heart disease prevention OR cardiovascular risk factors OR cardiovascular health outcomes OR coronary artery disease OR stroke OR hypertension OR atherosclerosis OR myocardial infarction) AND (public health interventions OR public health strategies OR nutritional epidemiology OR community health programs OR dietary guidelines OR health promotion) AND (randomized controlled trial OR case‒control study OR longitudinal study). Reference lists were manually searched to find any relevant work in the reference lists of the included studies.

### 2.1. Study Selection

Using the PICO framework, studies were selected based on the following criteria: population (P): individuals exposed to air pollution or environmental pollutants; intervention (I): dietary intake of flavonoids and flavonoid-rich food; comparison (C): not applicable; and outcome (O): cardiovascular disease risk mitigation and improvement in cardiovascular health outcomes related to air pollution exposure. Articles were eligible if they focused on this work’s main or specific objectives; were published between 2014 and 2024; were written in English; were observational, cohort, cross-sectional, a clinical trial, a narrative review, a systematic review, a meta-analysis, an umbrella analysis, or an analytical method; or were human, *in vitro*, or animal studies due to their crucial role in revealing the potential mechanisms of action and bioavailability of flavonoids on cardiovascular health.

Articles were excluded if they did not meet the specified criteria or were irrelevant to the research objectives, contained unpublished data, had sample sizes of less than 100 participants, were published before 2014, were duplicate publications, or were in languages other than English. The sample size criterion of 100 participants was selected to provide adequate statistical power and generalizability. Initial screening based on titles and abstracts was followed by a full-text review process conducted independently by three researchers. All discrepancies were resolved by discussion, achieving a 90% agreement rate.

### 2.2. Data Extraction and Synthesis

The following variables were extracted from the selected articles: air pollution and CVD, cardiovascular outcome/condition, air pollution exposure, and association, study design, and reference. For flavonoid studies: author, publication date, location, population, study design, flavonoid(s) studied, beneficial effects observed to prevent CVDs, limitations.

Then, content analysis confirmed the accuracy of the data used to identify thematic patterns [36]. Consistency in reported items was ensured through consensus between researchers. The data were synthesized and tabulated based on this consensus to create a complete matrix.

## 3. Results

### 3.1. Literature Retrieval Process and Basic Study Characteristics

**Figure 2:**
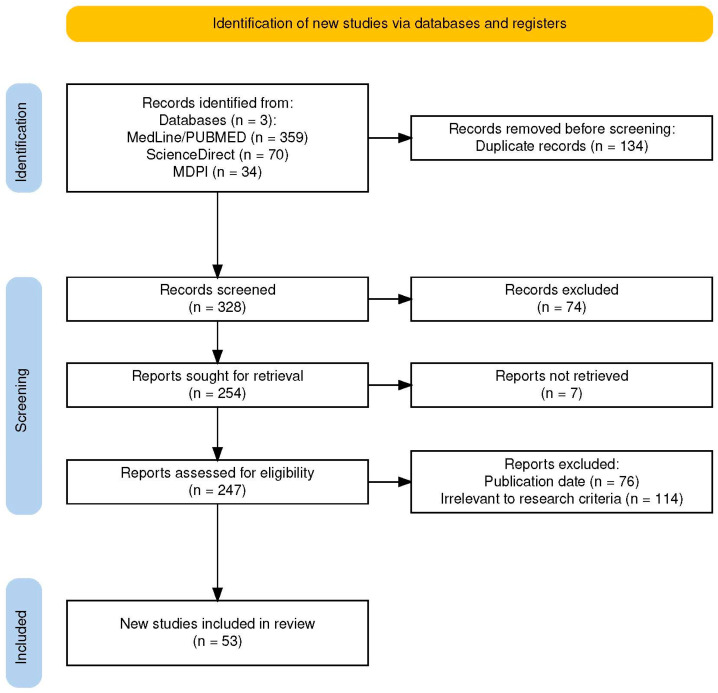
PRISMA flow diagram for the study selection and article eligibility process [49].

The literature search identified 463 articles from the databases, with 134 duplicate records removed. After screening and applying the eligibility criteria, 53 studies remained and were included in this narrative review (Figure 2). Basic study characteristics will be described separately according to the study’s scope to enhance clarity.

### 3.2. Studies on the Impact of Air Pollution on Cardiovascular Health

A total of 22 studies were included in this category. These studies were conducted in various locations, including 2 studies from the United States of America [24,50], 2 from Taiwan [7,51], 2 from China [52,53], 1 from the UK [23], and 15 from global or multiple locations [10,11,20,22,54,55,56,57,58,59,60,61,62,63,64]. The publication dates of the studies ranged from 2015 to 2024. Additionally, for the population studied, all studies were based on the general population, mainly adults, and two studies were conducted on adult patients [7,51].

The study designs included meta-analyses [11,54,58,63], systematic reviews [59,60], reviews [10,20,22,62,64], cohort studies [7,23,24,50,51,52], umbrella reviews [57,61], Mendelian randomization [56], and time-series studies [53].

Additionally, the focus of these studies varied. Some studies have investigated the associations between air pollution and general cardiovascular outcomes [23,24,50,52,53,55,56,57,59,60,63]. Other studies have examined air pollution and specific cardiovascular conditions, such as atrial fibrillation [54], heart failure [58], peripheral artery disease [62], atherosclerosis [20], blood pressure [11], and early cardiovascular disease [64]. Other studies have investigated environmental factors and cardiovascular health [22,61] and mechanistic studies on air pollution and cardiovascular effects [10]

The studies focused on specific air pollutant exposure and cardiovascular outcomes included 6 studies on PM2.5 [20,24,50,52,55,56], 3 studies on carbon monoxide [7,51,53], 10 on multiple pollutants [10,11,23,58,59,60,61,62,63,64], and 2 on environmental pollutants, including air pollutants [61,62]. Six studies measured long-term exposure [23,24,50,52,55,56]. Two studies investigated short-term exposure [53,54] and seven studies examined both long-term and short-term exposure [10,11,20,57,58,63,64].

### 3.3. Studies on the Cardiovascular Benefits of Dietary Flavonoids

The locations of the 31 studies included in this category varied: 8 from the United States of America [65,66,67,68,69,70,71,72], 2 from Denmark [73,74], 1 from France [75], 1 from Italy [76], 6 from multiple countries [77,78,79,80,81,82], and 12 for which the study location was not specified [66,83,84,85,86,87,88,89,90,91,92,93].

The study designs included 2 cross-sectional studies [67,72], 10 prospective cohort studies [65,68,69,70,71,73,74,75,76,94], 1 randomized controlled trial [87], 8 systematic reviews and meta-analyses [66,77,78,79,80,81,82,95], 9 literature reviews [83,84,85,86,88,89,90,91,92], and 1 narrative review [54].

These studies explored five main areas: (i) cardiovascular benefits of dietary flavonoids; eight studies focused on flavonoid intake and cardiovascular mortality [68,70,71,73,77,79,80,94]; nine studies on flavonoid intake and cardiovascular disease risk [65,66,69,75,76,78,81,82,88]; five studies on flavonoid intake and specific cardiovascular conditions [66,67,72,74,76]; (ii) mechanisms of flavonoid action; five studies investigated oxidative stress, inflammation, and other cardiovascular mechanisms [83,85,89,90,93]; (iii) dietary sources and patterns of flavonoids: one for flavonoid-rich fruits and vegetables [87], one for wine flavonoids [84], one for citrus flavonoids [91], and overall dietary flavonoid intake [65,66,67,68,70,72,73,74,75,76,77,78,79,80,81,82,94]; (iv) one study for flavonoid intake changes and health outcomes [71], and, finally, two studies on general health effects of flavonoids [91,92].

The selected studies also varied vastly in terms of the flavonoids studied, from major flavonoid subclasses [65,67,68,69,70,72,73,74,76,94] to flavonoid compounds [83,85,86,89,90,91,92,93], while other studies focused on flavonoid-rich foods [74,84,87]. Table 1 summarizes the characteristics of the selected studies on the impact of air pollution on cardiovascular health.

### 3.4. Impact of Air Pollution on Cardiovascular Health

- General cardiovascular outcomes

Research has shown diverse impacts of air pollution on general cardiovascular outcomes. One study attributed increased cardiovascular diseases (CVDs) to PM2.5 and O_3_ exposure [59]. Moreover, PM2.5, NO_2_, O_3_, and VOC pollutants were linked to increased occurrence of CVDs; acute effects such as myocardial infarction, coronary events and endothelial injury; and chronic processes such as atherosclerosis [22], while another study also linked pollutant exposure to the early onset of CVDs, specifically hypertension (HTN) and atherosclerosis [64]. A cohort study revealed that exposure to PM2.5, PM2.5 absorbance, PM10, NO_2_, and NOx is linked to the progression from pre-HTN to HTN (HR 1.105), CVD (HR 1.045), and death (HR 1.086) [23]. Chronic exposure to NO_2_, O_3_, PM10, PM2.5, and SO_2_ was also found to be associated with increased CVD incidence, hospitalization, disability, mortality, and costs [60]. Last, an increase in the burden of noncommunicable diseases (including CVD, stroke, ischemic heart disease, and coronary heart disease) was projected under adverse scenarios, such as growing population numbers, social deprivation, and an aging population [59].

- Specific cardiovascular conditions

In multiple studies, associations between air pollution and specific cardiovascular conditions were identified. Using Mendelian randomization [56], we observed a causal effect of PM2.5 on coronary artery disease (IVW: OR 2.06, 95% CI 1.35, 3.14). The same study [56] also revealed a causal effect of PM2.5 on HTN (IVW: OR 1.07, 95% CI 1.03, 1.12). A review reported that hypertension (HTN) was associated with chronic exposure to NO_2_, O_3_, PM10, PM2.5, and SO_2_ and was particularly linked to PM2.5 and NO_2_ [60]. A meta-analysis revealed that long-term exposure to PM2.5 (OR: 1.05) and short-term exposure to PM10, PM2.5, SO_2_, and NO_2_ (ORs: 1.05 to 1.10) are linked to HTN [11]. Additionally, a systematic review revealed that short-term exposure to PM2.5, PM10, and NOx was consistently associated with a high risk of HTN [57].

Regarding stroke, two studies reported similar results, showing that exposure to each 10 µg/m^3^ of PM2.5 increased the incidence of stroke by 13% [55] or 12% [63]. A systematic review revealed that short-term PM2.5, PM10, and NOx exposure was consistently associated with an increased risk of fatal and nonfatal stroke [57]. For MI, a cohort study revealed an 8% increased risk for each 10 µg/m^3^ of PM2.5 exposure [55,57]. A systematic review revealed associations between short-term exposure to PM2.5, PM10, and NOx (per 10-μg/m^3^ increase) and increased MI risk (1.20–2.40%, 95% CI 1.00–4.10%), PM10 (0.50–1.10%, 95% CI 0.10–1.60%), and NOx (1.10%, 95% CI 0.60–1.60%) [57].

Atrial fibrillation (AF): A meta-analysis revealed associations for both short-term and long-term exposure to air pollutants. For short-term exposure, AF attacks were associated with PM2.5, SO_2_, and NO_2_. The excess risk (ER) of an AF attack per 10 μg/m^3^ increase was 1.8% for PM2.5 and 1.1% for PM10. For gaseous pollutants, the ER per 10 parts per billion increase was 3.2% for NO_2_ and 2.9% for SO_2_. AF incidence was linked to long-term exposure to PM2.5, PM10, SO_2_, NO_2_, and CO [54]. Another meta-analysis focused on heart failure (HF) also reported associations; short-term exposure to PM2.5 (RR = 1.018) and PM10 (RR = 1.016) showed an increased risk and significant associations with NO_2_, SO_2_, and CO, while long-term exposure to various pollutants in the short-term is associated with HF [58].

For cardiac arrhythmia and other cardiac diseases, a review discussed the proarrhythmic effects of PM, lead, CO, SO_2_, and NOx, which are associated with ischemia, atrial, HF, and ventricular arrhythmias [10]. Regarding major adverse cardiovascular events (MACEs), a cohort study revealed that carbon monoxide poisoning was associated with a greater risk of MACEs (HR: 2.00, 95% CI: 1.83–2.18) [51]. Finally, a review reported that long-term exposure to PM10 is significantly associated with peripheral artery disease (PAD) (R^2^ = 0.5) [62]. Two studies confirmed that long-term exposure to PM2.5 is linked to both the development [20] and increased risk of atherosclerosis [57].

- Increased risk and mortality of CVD patients

Several studies have explored the impact of air pollution on cardiovascular mortality. A US cohort study revealed that each 10 μg/m^3^ increase in long-term PM2.5 exposure was linked to increased mortality from IHD (HR 1.16) and stroke (HR 1.14). Additionally, different levels of PM2.5 exposure (8–12 μg/m^3^, 12–20 μg/m^3^, and >20 μg/m^3^) were linked to increased mortality risks of 4%, 8%, and 19%, respectively [24]. A meta-analysis revealed an increase in mortality risk (RR: 1.10 to 1.33) per 10 μg/m^3^ increase in PM2.5 [50]. Another cohort study reported similar findings, associating the same incremental increase in PM2.5 levels with increased risks of IHD mortality (RR: 1.23) and cerebrovascular mortality (RR: 1.24). Additionally, a meta-analysis concluded that long-term exposure to PM2.5, PM10, NO_2_, and O_3_ is associated with an increased risk of CVD mortality (RRs: 1.11, 1.09, 1.23, and 1.03, respectively) [63].

Long-term exposure to PM2.5 even below the current US standards (12 µg/m^3^) was associated with an increased risk of CVD [24]. A meta-analysis provided strong evidence of increased risk with PM2.5, SO_2_, and NO_2_ [61]. A cohort study revealed that long-term exposure to PM2.5 (≥54 µg/m^3^) increases the risk of cerebrovascular disease (CeVD) [52]. CO poisoning was associated with an increased risk of arrhythmia (HR: 1.83) in a cohort study, and it was also highlighted that CO-poisoned patients with comorbidities have substantially increased risks of CVD [7]. A time-series study revealed that short-term exposure to ambient CO (per 1 mg/m^3^ increase) is associated with increased emergency room visits (ERVs) for total CVD (RR: 1.041) [53]. Table 2 summarizes the characteristics of the selected studies on the cardiovascular effects of flavonoids.

### 3.5. Cardiovascular Effects of Flavonoids

- Reduction in cardiovascular disease risk and mortality

Recent research has consistently demonstrated the beneficial effects of flavonoid intake on cardiovascular health. A higher intake of flavonoid-rich foods and beverages was found to be associated with a lower risk of cardiovascular mortality in a cohort of 55,786 females and 29,800 males [71]. Similarly, a cohort of 369,827 older adults reported that increased total flavonoid, flavonol, anthocyanidin, and flavone consumption was linked to reduced risks of death from CVD (HR 0.90–0.93), IHD (HR 0.89–0.94), CeVD (HR 0.84–0.89), and PAD (HR 0.79–0.81) [68]. Earlier cohort studies supported these findings, showing that moderate habitual flavonoid intake (~500 mg/d) [73] was inversely associated with CV mortality and CVD incidence. Furthermore, two studies highlighted the protective effects of quercetin and myricetin [70], as well as total flavonoids [69], on CVD-specific mortality. An inverse association between the intake of flavonoids, flavan-3-ols, anthocyanidins, and flavanones and cardiovascular risk, nonfatal events, and all-cause mortality was also observed [76]. Similarly, a cohort of 84,158 adults revealed a strong inverse association between CVD and the intake of every 10 mg/d of anthocyanins (HR 0.98), catechins (HR 0.98), and flavonols (HR 0.94) [75].

Moreover, review articles corroborate these findings: a 100 mg/d increase in flavonoid intake decreased CVD mortality risk by 4% [77]. Additionally, a 10 mg/d increase in flavonol intake decreased CVD risk by 5% [95] and emphasized the importance of diversified flavonoid subclasses and higher intake levels in reducing CVD risk [81,95]. In addition, a higher intake of kaempferol was linked to a decreased risk of CVD (RR 0.75, 95% CI: 0.56–1.00) [81]. High flavonoid intake was associated with reduced CVD mortality risk (pooled RR 0.86, 95% CI: 0.75, 0.98) [79]. A marginally significant association between total flavonoid intake and reduced CVD mortality was found (summary RR: 0.85; 95% CI: 0.70–1.03; *p* = 0.099) [80]. Finally, a meta-analysis specifically highlighted the benefits of flavan-3-ols in reducing CVD mortality by 13% [82].

- Effects on specific cardiovascular conditions

For hypertension (HTN), a cohort of 8010 adults exhibited a reduced risk of HTN with increased anthocyanin (OR 0.81, CI 0.66–0.99) and moderate flavan-3-ol (OR 0.79, CI 0.63–0.99) intake [72]. Furthermore, a cross-sectional study of 15,752 adults revealed a lower HTN risk for each unit increase flavonoid intake by 5% (OR: 0.95, 95% CI: 0.92–0.98) [67], while a cohort of 6110 adults with HTN showed that total flavonoid consumption (~375 mg/d) was linked to lower all-cause mortality in hypertensive patients [94]. For coronary heart disease (CHD), it was observed that a higher intake of anthocyanidin and proanthocyanidin was linked to a lower incidence of CHD by 29% (HR 0.71) and 37% (HR 0.63), respectively [65]. Similarly, increased flavonoid intake was associated with a reduced risk of CHD (summary RR 0.85, 95% CI: 0.79–0.91), while another study reported a 19% reduction in CHD incidence with increased habitual flavan-3-ol intake [82], while 12–14 mg/d quercetin intake was linked to a 28% reduced risk of CHD [81]. Finally, total flavonoid intake was linked to a reduced risk of CHDs (summary RR: 0.74; 95% CI: 0.54–1.02; *p* = 0.069) [80].

The findings revealed that an increase in flavonol intake of 20 mg/d was associated with a 14% reduction in stroke risk (summary RR 0.86, 95% CI: 0.75–0.99) [56]. For peripheral artery disease (PAD), a cohort study in Denmark with 55,647 adults revealed that the intake of 1000 mg/d of flavonoids compared to 174 mg/d (lowest median intake) was linked to a 32% lower risk of any PAD hospitalization (HR: 0.68; 95% CI: 0.60, 0.77) and a 47% lower risk of hospitalization for other PADs (HR: 0.53; 95% CI: 0.42, 0.67) [74]; similarly, a cohort of 369,827 older adults in the US reported that higher intakes of total flavonoids, flavonols, anthocyanidins, and flavones reduced PAD risk by approximately 19–21% (RR = 0.75, 95% CI: 0.56–1.00) compared to those with lower intake [68].

### 3.6. Mechanisms of Action and Evidence

The selected articles provided mechanistic insights into the impact of flavonoids on cardiovascular health.

- Endothelial function and vascular health

A randomized controlled trial with 174 adults at risk of CVD demonstrated significant improvements. A high-flavonoid fruit and vegetable diet (>15 mg/100 g of total flavonoids) improved endothelium-dependent microvascular reactivity (*p* = 0.017) in men with the addition of +2 portions/day after 6 weeks [87]. Additionally, flavanol-rich cocoa improved flow-mediated dilation and reduced blood pressure [88], while notable improvements in vascular function and blood pressure regulation were found [82]. The administration of hesperidin to humans and animals also restored endothelial function [93]. Moreover, a high-flavonoid diet with an increase of +4 portions per day after 12 weeks significantly increased plasma nitric oxide (NO) levels (*p* = 0.0243) in the whole study group [87]. Maalik et al. highlighted that flavonoids such as quercetin, kaempferol, epicatechin, daidzein, and hesperetin increase nitric oxide (NO) levels. They achieve this by directly stimulating endothelial nitric oxide synthase (eNOS), activating the PI3K/Akt pathway to enhance eNOS activity through phosphorylation, and increasing intracellular calcium levels, which promote NO synthesis [86]. The role of anthocyanins and hesperetin in vasodilation through the modulation of K^+^ and Ca^2+^ ion channels has also been emphasized [83].

- Antioxidant effects

Flavonoids, including catechins, epicatechins, procyanidins, and herbal monomers such as baicalin, quercetin, luteolin, and naringin, exhibit antioxidant properties through multiple mechanisms [84,90]. These compounds act as electron donors, neutralizing reactive oxygen species (ROS), such as peroxynitrite, hydroxyl, and peroxyl radicals [83]. They modulate and enhance antioxidant defense systems, preventing oxidative stress and reducing oxidative damage [84]. Flavonoids interact with enzymes involved in ROS production, including glutathione S-transferase, NADH oxidase, microsomal monooxygenase, and mitochondrial succinoxidase [83]. Quercetin can chelate free metal ions, reducing free radical formation [83], and has been shown to increase glutathione levels and superoxide dismutase activity [83,90], which could directly counteract the oxidative effects of pollutants. The antioxidant effects of flavonoids include direct ROS scavenging, metal ion chelation, lipid peroxidation reduction, and enhancement of antioxidant systems by modulating the Nrf2/HO-1 pathway [85,90]. This modulation increases mitochondrial enzyme activity and upregulates antioxidant enzymes [90]. Hesperidin supplementation has been shown to increase total antioxidant capacity compared to that in placebo groups [93].

- Anti-inflammatory effects

A high-flavonoid fruits and vegetables diet with +2 portions/day after 6 weeks reduced C-reactive protein (*p* = 0.001) and E-selectin (*p* =0.0005) in men [87]. A total of 320 mg/d twice a day for six months in hypercholesterolemic individuals decreased C-reactive protein, soluble vascular cell adhesion molecule-1, and plasma IL-1β [92]. Hesperidin led to notable decreases in the levels of TNF-α and IL-6 in type 2 diabetes patients, while a hesperidin-derived metabolite suppressed endothelial cell inflammation [93]. Red wine flavonoids have been demonstrated to inhibit the biosynthesis of eicosanoids, particularly cyclooxygenases and 5-lipoxygenase enzymes. These enzymes metabolize free arachidonic acid to produce proinflammatory compounds, which are known to initiate the inflammatory cascade [84]. Wine flavonoids can activate the NF-kB pathway, reducing the release of proinflammatory cytokines [84]. A study revealed that increased flavonoid intake was associated with decreased methylation of the TLR2 gene, a principal player in the inflammatory response [96]. Additionally, several flavonoids inhibit the TLR4-NF-kB and PI3K-AKT pathways to reduce inflammatory cytokine production [85,90]. The main flavonoid subgroups show anti-inflammatory potential by modulating eicosanoid and prostaglandin synthesis, inhibiting neutrophil degranulation, reducing the concentration of arachidonic acid, and inhibiting phosphodiesterase.

- Blood pressure regulation

Flavonoids indirectly contribute to blood pressure (BP) regulation by improving endothelial function and modulating the renin–angiotensin–aldosterone system [92]. Specifically, catechins from cocoa have been associated with lower systolic (SBP) and diastolic (DBP) blood pressure in both human and animal studies. Additionally, epicatechin has demonstrated a mean reduction of 4.1 and 2.0 mmHg in SBP and DBP, respectively [86]. Catechin-rich green tea decreased SBP and DBP [93]. For hypertension, it is well documented that hesperetin and naringin have antihypertensive effects; likewise, the antihypertensive effect of anthocyanins is more pronounced in subjects with higher BP [86]. Rutin and quercetin also demonstrated antihypertensive effects after retarding the effects of a high-salt diet in rats [93]. Finally, various *in vitro* and rat studies have shown the antihypertensive capacity of kaempferol, quercetin, naringenin, and epicatechin [83]. The consumption of flavonoid-rich cocoa (450–900 mg/day over one week to one month) resulted in a reduction in BP, particularly in individuals with compromised vascular health [88].

- Antiplatelet and antithrombotic effects

Evidence shows that flavonoids have antiplatelet and antithrombotic effects through multiple mechanisms. Wine flavonoids, for instance, inhibit platelet aggregation by altering membrane fluidity, ligand‒receptor affinity, and intracellular signaling pathways and even mediating antiplatelet effects through antioxidant and NO pathways [84]. Various flavonoids, including quercetin, kaempferol, proanthocyanidins, naringenin, formononetin, nobiletin, anthocyanins, vitexin, and calycosin, demonstrate these effects. They act by reducing collagen-induced aggregation, inhibiting thromboxane A2 production, regulating lipid metabolism, reducing inflammation, enhancing antioxidant activity, promoting autophagy [85], and reducing the progression of atheroma plaques [83]. Notably, 500 mg/day of quercetin attenuates atherosclerosis, while 50 or 100 mg/kg/day of kaempferol in mice decreases the atherosclerotic lesion area and improves vasorelaxation [92]. Furthermore, flavonoids reduce the risk of atherosclerosis and atherothrombotic disease by inhibiting excessive tissue factor availability in the endothelium [89].

- Cardioprotective effects

Two studies underscore the effect of flavonoids in modulating the PI3K-AKT pathway, which is known to improve endothelial function and vasodilation and reduce oxidative stress, thereby aiding cardiovascular health [85,86]. Additionally, flavonoids influence the TLR4-NF-κB pathway [85], which can mitigate inflammation and protect against cardiovascular damage. Flavonoids modulate inflammation, reducing cardiomyocyte death and minimizing postischemic infarct size in rats [78]. Low concentrations of epigallocatechin-3-gallate in green tea increased the survival of myocytes [93].

- Lipid metabolism

The administration of rutin and quercetin increased lipid peroxidation, retarding the increase in triglyceride, LDL, and total cholesterol serum levels induced by a high-salt diet, while catechin-rich green tea produced an overall restoration to normal levels in the lipid profile, specifically an important decrease in LDL cholesterol [93]. Raman et al. reported that flavan-3-ols significantly decreased TGs (net change: −0.03 mmol/L), LDL (net change: −0.07 mmol/L), and total cholesterol (net change: −0.14 mmol/L) while significantly increasing HDL (net change: 0.03 mmol/L) [82]. Daily oral intake of 450 mg of naringin for 90 days had effects on obese patients with hypercholesterolemia, reduced body weight, and reduced lipid profiles [91]. Similarly, the administration of 270 mg of flavonoid-rich citrus fruit reduced cholesterol (20–30%) and LDL (19–27%) within four weeks [92].

- Glucose and insulin metabolism

The results showed that flavan-3-ols significantly decreased hemoglobin A1c levels (27 RCTs, net change: −0.05%; 95% CI: −0.09%, −0.01%; I2 = 0.0%) and HOMA-IR (35 RCTs, net change: −0.15, with a 95% CI of −0.29 to −0.01) [82].

## 4. Discussion

### 4.1. Air Pollution and Cardiovascular Risks

The evidence presented in this work demonstrates the significant and wide-ranging impacts of air pollution on cardiovascular health. Consistent associations have been observed between various air pollutants, particularly PM2.5, PM10, NO_2_, O_3_, and SO_2_, and increased risks of CVDs or associated risk factors. These include endothelial injury and systemic inflammation, HTN, stroke, MI, AF, and HF [18,22,59,64].

Short-term exposure to air pollutants has been linked to acute cardiovascular events and mortality [5,22,54,57]. Meanwhile, long-term exposure to air pollutants has been consistently associated with the development and progression of chronic cardiovascular conditions. Studies have demonstrated links between PM2.5 exposure and increased risks of hypertension, cardiovascular disease, and mortality [23]. Causal relationships have been established between PM2.5 exposure and both coronary artery disease and hypertension using Mendelian randomization techniques [56]. These findings are corroborated by earlier research indicating that long-term PM2.5 exposure significantly exacerbates cardiovascular risk and reduces life expectancy [6].

The evidence suggests that air pollution contributes to the development of atherosclerosis, endothelial dysfunction, and systemic inflammation, which are major pathophysiological mechanisms in cardiovascular diseases [18,20,57]. These processes may explain the observed increases in both acute events and chronic disease progression.

Moreover, multiple studies indicate that exposure to air pollutants increases the risk of inflammatory response, oxidative stress, cardiovascular mortality, and disability, even at levels below current regulatory standards in some countries [20,21,24]. This underscores the importance of reducing air pollution levels as much as possible to protect public health, noting that short-term elevations in PM2.5 have been shown to increase the relative risk of acute cardiovascular events by 1% to 3%, while long-term exposures increase this risk by approximately 10% [21].

Notably, the effects of air pollution on cardiovascular health may be compounded by other environmental and lifestyle factors. Carbon monoxide poisoning has been associated with a greater risk of major adverse cardiovascular events [51], highlighting the potential for synergistic effects between different types of environmental exposure. Furthermore, coexposure to air and noise pollution, which are prevalent in modern urban societies, may contribute to the development of hypertension and diabetes mellitus [97].

Given the significant cardiovascular risks posed by air pollution, there is a pressing need for strategies to mitigate these effects. While reducing air pollution levels remains the primary goal, complementary approaches to protect cardiovascular health are also crucial. Dietary interventions, particularly those rich in bioactive compounds such as flavonoids, have emerged as a promising area of research. The following section explores the cardiovascular benefits of flavonoids and their potential to counteract the harmful effects of air pollution.

### 4.2. Cardiovascular Benefits of Dietary Flavonoids

Research on flavonoids has shown significant benefits for cardiovascular health, ranging from a reduced risk of CVD to improvements in specific conditions [68,71,73,77]. Specific flavonoids such as quercetin, myricetin, and kaempferol have been highlighted for their protective effects against CVD-specific mortality [69,70,81].

Different subclasses of flavonoids offer various cardiovascular benefits. Anthocyanins and flavan-3-ols have been linked to a reduced risk of hypertension [67,72] and a lower incidence of coronary heart disease (CHD) [65,82]. In contrast, flavonols have shown promise in reducing overall CVD [95] and stroke [66] risk. Proanthocyanidins have also demonstrated significant benefits, such as lower incidence of CHD [65].

The cardiovascular benefits of flavonoids are supported by multiple mechanisms of action. They have been shown to improve endothelial function by enhancing endothelium-dependent microvascular reactivity and increasing NO levels [86,87]. Flavonoids also exhibit potent antioxidant effects, neutralizing reactive oxygen species and boosting antioxidant defense systems [83,84,90] by modulating the Nrf2/HO-1 pathway [85,90]. The anti-inflammatory properties of these compounds include their ability to modulate the TLR4-NF-ĸB and PI3K-AKT pathways [85,90], reduce inflammatory cytokine production, and subsequently decrease inflammatory markers such as C-reactive protein and E-selectin [87,92].

Furthermore, flavonoids play a crucial role in blood pressure regulation. Catechins and epicatechin, in particular, have demonstrated antihypertensive effects [86,93]. The antiplatelet and antithrombotic effects of flavonoids, including their ability to inhibit platelet aggregation and reduce the risk of atherosclerosis, have been well documented [84,85,89].

Last, flavonoids not only positively influence lipid metabolism by improving lipid profiles, such as reducing LDL cholesterol and triglycerides while increasing HDL cholesterol [82,93], but also exert beneficial effects on glucose and insulin metabolism. For instance, flavan-3-ols have been shown to significantly decrease hemoglobin A1c levels and improve insulin resistance [82].

Strong evidence supports the ability of flavonoids to positively impact multiple aspects of cardiovascular health through various mechanisms, underscoring their potential as a valuable dietary component in the prevention and management of cardiovascular diseases.

Direct comparisons among these flavonoid subclasses suggest that while all offer cardiovascular benefits, their effects may vary significantly. For instance, flavonols and proanthocyanidins appear particularly effective in reducing CVD and stroke risk, respectively, compared to other flavonoids. Catechins and epicatechins also show notable benefits for blood pressure regulation and endothelial function. However, more direct comparisons between the effects of different types of flavonoids on cardiovascular health are needed. Further research in this area could provide more clarity on their relative benefits and help refine dietary recommendations.

### 4.3. Potential Interactions between Flavonoid Intake and Air Pollution Exposure

Based on the evidence reviewed, we hypothesize that dietary flavonoids could mitigate and prevent the adverse cardiovascular effects of air pollution exposure through several mechanisms. Although not explicitly discussed in our review of air pollution and cardiovascular health, oxidative stress is a key underlying mechanism of air pollution-induced cardiovascular damage.

We propose the following specific mechanisms by which flavonoids may mitigate the cardiovascular effects of air pollution:

#### 4.3.1. Improvement in Endothelial Function Impaired by Pollutants

Endothelial dysfunction, a critical factor in cardiovascular risk, is a well-documented consequence of exposure to airborne particulate matter [6,18,22]. Flavonoids enhance NO levels by stimulating eNOS and activating the PI3K/Akt pathway, along with increasing intracellular calcium levels [86]. These mechanisms improve endothelium-dependent vasodilation and may counteract pollution-induced endothelial dysfunction [83,87,93].

#### 4.3.2. Antioxidant Effects

The cardiovascular system is particularly vulnerable to oxidative stress induced by air pollutants, especially particulate matter [17,21,22,61]. Flavonoids exhibit potent antioxidant properties, scavenge free radicals, and upregulate antioxidant systems [83,85,90].

#### 4.3.3. Anti-Inflammatory Actions

Systemic inflammation, a main factor in cardiovascular disease, is significantly exacerbated by elevated levels of air pollution [20,52]. Flavonoids exhibit anti-inflammatory effects through multiple pathways, including inhibiting proinflammatory cytokines, decreased methylation of the TLR2 gene, and modulating signaling pathways such as the TNF-κB, TLR4-NF-ĸB, and PI3K-AKT pathways [84,85,90,93,96].

#### 4.3.4. Blood Pressure Regulation to Offset the Hypertensive Effects of Pollution

Hypertension, a major risk factor for cardiovascular disease, has been consistently linked to air pollution exposure [6,11,21,56,60]. Flavonoids have shown antihypertensive effects in numerous studies [67,72,94]. The mechanisms include the modulation of endothelial eNOS, reduction of oxidative stress, and improvement of vascular reactivity [86].

#### 4.3.5. Antiplatelet and Antithrombotic Effects Mitigating Pollution-Induced Thrombosis Risk

Long-term exposure to high levels of air pollution, especially particulate matter, increases platelet activation and thrombotic risk [20,21,61]. Flavonoids demonstrate significant antiplatelet and antithrombotic effects by inhibiting platelet aggregation, suppressing phospholipase C and protein kinase C phosphorylation, and modulating calcium mobilization [85,89]. They also reduce tissue factor expression and attenuate atherosclerosis development by decreasing oxidized LDL levels [83,85].

Although direct studies examining these interactions are limited, the established cardiovascular benefits of flavonoids and their ability to counteract the underlying mechanisms of pollution-induced damage provide a strong rationale for the hypothesis that flavonoid intake may help mitigate the cardiovascular risks associated with air pollution exposure. Further research directly examining this interaction is warranted. Other studies have shown how flavonoids protect reproductive health and counteract cancer [98], and due to similar mechanisms, air pollutants, triggering cardiovascular health protection from air pollution-induced risks.

### 4.4. Public Health Implications

Complementary public health measures are essential considering the widespread exposure to air pollution and the challenges in rapidly reducing emissions. Flavonoids have significant potential to mitigate air pollution-induced cardiovascular risks, making dietary interventions a promising strategy for protecting cardiovascular health.

Several strategies to mitigate the cardiovascular effects of air pollution, including personal, local, public health policy, and research-based interventions [21,99], must be used for a better approach. Based on our aims and scope, we will focus on the strategies most relevant to promoting dietary flavonoids as a complementary approach while also briefly mentioning common strategies to reduce air pollution.

These interventions include encouraging and facilitating research to explore flavonoid bioavailability, mechanisms of action [35,85,86,89,90,91,92], and complementary strategies, including dietary interventions, to mitigate the cardiovascular impacts of air pollution [98,100,101,102]. Additionally, increasing public awareness about the health risks of air pollution [103,104] and the benefits of dietary interventions to reduce the negative health impacts of air pollution [105,106,107,108,109] is essential. Promoting the consumption of flavonoid-rich diets and considering supplementation, when necessary, while ensuring accessibility regardless of socioeconomic status or geographic location [106,110] is also important. Common strategies for reducing air pollution include transitioning to cleaner and renewable energy sources, implementing land use assessments, relocating major traffic sources, promoting low-emission and zero-emission vehicles, encouraging active transport, and adopting sustainable agricultural practices [108,111].

However, it is crucial to emphasize that while dietary interventions, such as increased flavonoid intake, can offer additional protection, they should not replace efforts to reduce air pollution. Instead, they should be seen as a complementary strategy to protect public health, while broader and sustainable efforts to improve air quality are pursued [112]. The primary focus must remain on reducing air pollution levels through stricter regulations and cleaner technologies [113]. By implementing these actions, we can potentially offer an additional layer of protection for cardiovascular health while still addressing the core cause of air pollution.

### 4.5. Limitations and Strengths

Despite the evidence suggesting promising ways for dietary flavonoids to mitigate cardiovascular disease risks associated with air pollution exposure, several limitations are worthy of consideration.

First, the variability in bioavailability among different flavonoids presents a significant challenge in assessing their health effects. Many studies rely on *in vitro* experiments that use doses higher than those typically bioavailable in humans, complicating the translation of these findings into real-world efficacy. This discrepancy, coupled with inconsistencies in bioavailability influenced by factors such as age, metabolism, and the complexity of flavonoid chemistry, obscures the true impact of flavonoids on health.

Another important issue related to flavonoid intake is the fact that there is no consensus on what is considered a moderate or high intake, and inconsistent values appear throughout the reviewed studies. Furthermore, dietary flavonoid intake is often assessed only at baseline, which may not accurately reflect subsequent dietary changes and could introduce recall bias. Self-reported dietary intake methods, commonly used in studies, are prone to inaccuracies and recall bias, potentially skewing the results. Thus, while *in vitro* studies offer valuable insights, further research is crucial to bridge the gap between laboratory findings and actual physiological responses and to refine dietary recommendations.

Second, the study designs predominantly utilized in the reviewed articles limit causal inference. Observational designs and meta-analyses cannot definitively establish cause-and-effect relationships, and potential residual confounding factors, such as unadjusted total energy intake or lifestyle changes postdiagnosis, may have influenced the outcomes. The heterogeneity in dietary assessment methods and the variability in flavonoid composition between different foods and beverages further complicate the interpretation of the results. Additionally, some studies did not stratify for hypertension severity or account for changes in dietary data over extended periods, affecting the detection of associations and limiting generalizability.

Despite these limitations, this narrative review has several strengths. This study provides a comprehensive synthesis of the literature on the intersection of dietary flavonoids and cardiovascular health in the context of air pollution exposure, highlighting a novel and underexplored area of public health research. By gathering evidence from diverse studies, this review underscores the potential of flavonoids to counteract the underlying mechanisms of pollution-induced cardiovascular damage, providing a strong rationale for further investigation.

Finally, we emphasize the importance of dietary interventions as a complementary strategy to broaden efforts aimed at reducing the impact of air pollution on human health. By integrating findings from both clinical trials and epidemiological studies, this study presents a balanced perspective on the feasibility and potential impact of promoting flavonoid-rich diets. This approach supports ongoing research and advocates for public health initiatives to raise awareness and encourage the consumption of flavonoid-rich foods as part of a holistic strategy to protect cardiovascular health.

## 5. Conclusions

The evidence highlights the significant cardiovascular risks posed by air pollution and the potential use of dietary flavonoids to mitigate these effects. Air pollutants such as PM2.5, PM10, NO_2_, O_3_, and SO_2_ are linked to increased risks of hypertension, stroke, myocardial infarction, atrial fibrillation, and heart failure. Flavonoids, with their antioxidant, anti-inflammatory, and endothelial-function-improving properties, could counteract the cardiovascular damage caused by these pollutants.

Although reducing air pollution remains the primary goal, promoting flavonoid-rich diets is a promising complementary strategy to protect cardiovascular health. Public health initiatives should focus on raising awareness about the benefits of flavonoids and encouraging their consumption as part of an integral approach to mitigate the cardiovascular impacts of air pollution.

Further research is needed to explore the direct interactions between flavonoid intake and air pollution exposure. However, the current evidence supports the integration of dietary interventions into broader public health strategies to reduce the health impacts of air pollution on cardiovascular health.

## Figures and Tables

**Table 1:** Summarized findings on the association between air pollution exposure and cardiovascular outcomes.

Cardiovascular Outcome/Condition	Air Pollution Exposure and Association	Study Design	Author
**General Cardiovascular Outcomes**			
Increased occurrence of CVDs	Increased exposure to PM2.5 and O_3_ (CHD, IHD, and stroke)	Systematic review	Karim, 2024 [59]
	PM2.5, NO_2_, O_3_, and VOC associations: short-term outcomes, acute effects (myocardial infraction, coronary events, endothelial injury) and long-term exposure, chronic processes (atherosclerosis)	Review	Blaustein et al., 2024 [22]
Early onset of CVDs	PM2.5, NO_2_, O_3_, and VOC, specifically HTN and atherosclerosis	Review	Zhang et al., 2023 [64]
Progression from pre-HTN to HTN, CVD, and death	Exposure to PM2.5 associated with progression from pre-HTN to HTN (HR: 1.105), pre-HTN to CVD (HR: 1.045), and pre-HTN to death (HR: 1.086). Air pollution could act directly on CVD progression	Cohort study	Zhang et al., 2023 [23]
Increased prevalence, hospitalization, disability, mortality, and costs of CVDs	Chronic exposure to NO_2_, O_3_, PM10, PM2.5, and SO_2_	Review	Khoshakhlagh, 2024 [60]
Projected increase in NCD burden	Projected increase in CVD, stroke, IHD, and CHD under adverse scenarios	Systematic review	Karim et al., 2024 [59]
**Specific cardiovascular conditions**			
Coronary artery disease	Causal effect observed with PM2.5 (IVW: OR 2.06, 95% CI 1.35, 3.14)	Mendelian randomization	Cao et al., 2024 [56]
Hypertension (HTN)	Causal effect observed with PM2.5 (IVW: OR 1.07, 95% CI 1.03, 1.12)	Mendelian randomization	Cao et al., 2024 [56]
	Chronic exposure to NO_2_, O_3_, PM10, PM2.5, and SO_2_, with PM2.5 and NO_2_ being closely associated	Review	Khoshakhlagh, 2024 [60]
	Long-term exposure to PM2.5 (OR: 1.05); short-term exposure to PM10, PM2.5, SO_2_, and NO_2_ (ORs: 1.05 to 1.10)	Meta-analysis	Yang et al., 2018 [11]
	Short-term exposures to PM2.5, PM10, and NOx consistently associated with increased risks of HTN	Systematic review	de Bont et al., 2022 [57]
Stroke	Long-term exposure to PM2.5 (per 10 µg/m^3^ increase) associated with a 13% increased risk	Cohort study	Alexeeff et al., 2021 [43]
	Long-term exposure to PM2.5 (per 10 μg/m^3^ increase) associated with increased risk (RR: 1.12, 95% CI 1.05–1.19)	Meta-analysis	Yang et al., 2019 [63]
	Short-term exposures to PM2.5, PM10, and NOx consistently associated with increased risks of stroke (fatal and nonfatal)	Systematic review	de Bont et al., 2022 [57]
Incident myocardial infarction (MI)	Long-term exposure to PM2.5 (per 10 µg/m^3^ increase) associated with an 8% increased risk	Cohort study	Alexeeff et al., 2021 [55]
	Short-term exposure to PM2.5, PM10, and NOx (per 10 μg/m^3^ increase) is associated with increased MI risk: PM2.5 (1.20–2.40%, 95% CI 1.00–4.10%), PM10 (0.50–1.10%, 95% CI 0.10–1.60%), and NOx (1.10%, 95% CI 0.60–1.60%)	Systematic review	de Bont et al., 2022 [57]
Atrial fibrillation (AF)	AF attack: Short-term exposure to PM2.5 (ER: 1.8%), SO_2_ (ER: 2.9%), and NO_2_ (ER: 3.2%) per 10 μg/m^3^ or 10 ppb increase AF incidence: Long-term exposure to PM2.5, PM10, SO_2_, NO_2_, and CO	Meta-analysis	Chen et al., 2021 [54]
Heart failure (HF)	Short-term exposure: PM2.5 (RR: 1.018), PM10 (RR: 1.016), and significantly associated with NO_2_, SO_2_, CO Long-term exposure: PM2.5 (RR: 1.748), PM10 (RR: 1.212), NO_2_ (RR: 1.204)	Meta-analysis	Jia et al., 2023 [58]
Cardiac arrhythmias and other cardiac diseases	PM, lead, CO, SO_2_, and NOx proarrhythmic effects associated with HF, ischemia, atrial and ventricular arrhythmias	Review	Palacio et al., 2023 [10]
Major adverse cardiovascular events (MACEs)	CO poisoning associated with higher risk of MACEs (HR: 2.00, 95% CI: 1.83–2.18)	Cohort study	Wong, 2017 [51]
Peripheral artery disease (PAD)	Long-term exposure to PM10 significantly associated with PAD (R^2^: 0.5)	Review	Serra et al., 2021 [62]
Atherosclerosis	Long-term exposure to PM2.5 associated with the development of atherosclerosis	Review	Tian et al., 2021 [20]
	Long-term exposure to PM2.5 associated with increased risk of atherosclerosis	Systematic review	de Bont et al., 2022 [57]
**Increased Risk and Mortality of CVDs**			
Increased risk of mortality from CVDs (including IHD and stroke)	Long-term exposure to PM2.5 (per 10 μg/m^3^ increase), IHD mortality increased (HR: 1.16), stroke mortality increase (HR: 1.14) Different levels of PM2.5 exposure, 8–12 μg/m^3^, 12–20 μg/m^3^, and >20 μg/m^3^, are associated with increased mortality risk by 4%, 8%, and 19%	Cohort study	Hayes, 2020 [24]
	Long-term exposure to PM2.5 (per 10 μg/m^3^ increase) associated with increased mortality risk (RR: 1.10 to 1.33)	Meta-analysis	Pun, 2017 [50]
	Long-term exposure to PM2.5 (per 10 μg/m^3^ increase) associated with increased risks of IHD mortality (RR: 1.23) and cerebrovascular mortality (RR: 1.24)	Cohort study	Alexeeff et al., 2021 [55]
	Long-term exposure to PM2.5, PM10, NO_2_, and O_3_ associated with increased risk of CVD mortality (RRs: 1.11, 1.09, 1.23, and 1.03, respectively)	Meta-analysis	Yang et al., 2019 [63]
Increased risk of CVD	Long-term exposure to PM2.5 below the current US standards (12 µg/m^3^)	Cohort study	Hayes, 2020 [24]
	Strong evidence of increased risk with PM2.5, SO_2_, NO_2_ exposure	Meta-analysis	Liu et al., 2023 [61]
	Long-term exposure to PM2.5 (≥54 µg/m^3^) increases CeVD risk	Cohort study	Sun et al., 2023 [52]
	CO poisoning associated with increased risk of arrhythmia (HR: 1.83). CO-poisoned patients with comorbidities have substantially increased risk of CVD	Cohort study	Lee et al., 2015 [7]
	Short-term exposure to ambient CO (per 1 mg/m^3^ increase) associated with increased ERVs for total CVD (RR: 1.041)	Time-series study	You et al., 2023 [53]

CVD: Cardiovascular disease; PM2.5: Particulate matter with a diameter < 2.5 μm; PM10: Particulate matter with a diameter < 10 μm; IHD: Ischemic heart disease; NO_2_: Nitrogen dioxide; O_3_: Ozone; SO_2_: Sulfur dioxide; CeVD: Cerebrovascular disease; HTN: Hypertension; NCD: Noncommunicable disease; CHD: Coronary heart disease; AF: Atrial fibrillation; MACE: Major adverse cardiovascular event; PAD: Peripheral artery disease; MI: Myocardial infarction; HF: Heart failure; CO: Carbon monoxide; NOx: Nitrogen oxides; ERVs: Emergency room visits; OR: Odds ratio; RR: Risk ratio; HR: Hazard ratio; CI: Confidence interval; IVW: Inverse variance weighted.

**Table 2:** Summarized findings of flavonoid effects on cardiovascular health.

Original articles							
Author	Publication Date	Location	Population	Study Design	Flavonoid(s) Studied	Beneficial Effects Observed to Prevent CVDs	Limitations
Wan et al. [72]	2024	United States	8010 adults	Cross-sectional analysis of NHANES (2007–2010 and 2017–2018)	29 flavonoid compounds and main subgroups (anthocyanidins, flavan-3-ols, flavanones, flavones, flavonols, and isoflavones)	Reduced risk of hypertension with higher anthocyanin intake (>12.904 mg/day, from diet) (OR: 0.81; 95% CI: 0.66–0.99) and moderate flavan-3-ol intake (16.895–170.916 mg/day, from diet) (OR: 0.79; 95% CI: 0.63–0.99).	Study design limits causal inference. Dietary flavonoid intake recall bias.
Hu et al. [67]	2024	United States	15,752 adults	Cross-sectional study (NHANES data from 2007–2010 and 2017–2018)	29 flavonoid compounds and main subgroups (excluding isoflavones)	Higher total flavonoid intake (specific mg/day not detailed in the data, from diet) was significantly associated with a lower risk of hypertension (OR: 0.78; 95% CI: 0.70–0.88). Additionally, for each unit increase in flavonoid intake (specific mg/day not detailed in the data, from diet), the risk of hypertension decreased by 5% (OR: 0.95; 95% CI: 0.92–0.98).	Study design limits causal inference. HTN severities were not stratified. Potential biases from lifestyle changes postdiagnosis. Flavonoid intake was assessed only at baseline.
Wang Kan et al. [94]	2024	United States	6110 adults with HTN	Prospective cohort study (NHANES data from 2007–2008, 2009–2010, and 2017–2018)	29 flavonoid compounds and main subgroups (anthocyanidins, flavan-3-ols, flavanones, flavones, flavonols, and isoflavones)	Reduced risk of all-cause mortality with higher total flavonoid intake (~375 mg/day, from diet) was observed in hypertensive patients (HR: 0.74; 95% CI: 0.56–0.97).	Study design limits causal inference. Possible misclassification of dietary habits.
Bondonno et al. [71]	2023	United States	55,786 females and 29,800 males	Cohort study (NHS and HPFS data from 1994–2018)	Flavonoid-rich foods and beverages (blueberry, apple, orange, tea, onion, pepper, raisin, red wine, orange and grapefruit juice)	Increased intake of flavonoid-rich foods for each 3.5 servings/week increase of blueberries (HR: 0.95; 95% CI: 0.91–0.99), red wine (HR: 0.96; 95% CI: 0.93–0.99), and peppers (HR: 0.91; 95% CI: 0.88–0.95) showed a 4–9% lower cardiovascular mortality risk.	Variability in the flavonoid composition between the seven foods and beverages in flavodiet score. Possible residual confounding despite adjustments. Study design limits causal inference.
Zhao et al. [68]	2023	United States	369,827 older adults	Prospective cohort study (NIH-AARP Diet and Health Study)	Total flavonoids and main subgroups (flavonols, flavanols, flavanones, flavones, anthocyanins)	Higher intake from diet of total flavonoids (660 mg/day), flavonols (37.8 mg/day), anthocyanidins (26.9 mg/day), and flavones (2.8 mg/day) is linked to lower risks of death from CVDs (HR: 0.90–0.93), IHD (HR: 0.89–0.94), CeVD (HR: 0.84–0.89), and PAD (HR: 0.79–0.81).	Self-reported dietary intake may lead to recall bias. Baseline flavonoid intake may not accurately reflect subsequent dietary changes. Possible residual confounding despite adjustments. Study design limits causal inference.
Bondonno et al. [73]	2019	Denmark	56,048 adults	Prospective cohort study (Danish Diet, Cancer, and Health cohort)	Total flavonoids and main subgroups (flavonols, flavanols, flavanones, flavones, anthocyanins)	Moderate habitual flavonoid intake (~500 mg/day, from diet) inversely associated with cardiovascular mortality (P-nonlinearity < 0.001), particularly in smokers and heavy drinkers.	Study design limits causal inference. Dietary data may have changed over 23 years, affecting association detection. Limited generalizability due to homogeneity of population.
Bondonno et al. [74]	2021	Denmark	55,647 adults	Prospective cohort study (Danish Diet, Cancer, and Health cohort)	Total flavonoids and main subgroups (isoflavones were excluded)	Higher total flavonoid intake (~750–1000 mg/d) vs. lowest median intake (174 mg/d) linked to significantly lower risks of any PAD hospitalization (HR: 0.68; 95% CI: 0.60–0.77), atherosclerosis (HR: 0.74; 95% CI: 0.62–0.88), aneurysm (HR: 0.72; 95% CI: 0.59–0.88), hospitalization for other PADs (HR: 0.53; 95% CI: 0.42–0.67).	Study design limits causal inference. Dietary data may have changed over 23 years, affecting association detection. Limited generalizability due to homogeneity of population.
Goetz et al. [65]	2016	United States	16,678 adults	Prospective cohort study (REGARDS study, 2003–2007)	Total flavonoid, anthocyanidins, flavan-3-ols, flavanones, flavone, flavonols, and proanthocyanidins	Higher intake of anthocyanidin (≥18.6 mg/day, from diet) (HR: 0.71; 95% CI: 0.52–0.98) and proanthocyanidin (≥137 mg/day, form diet) (HR: 0.63; 95% CI: 0.47–0.84) linked to lower incidence of CHD.	Self-reported dietary intake may lead to recall bias. Limited by the accuracy of the USDA flavonoid database.
Macready et al. [87]	2014	United Kingdom	174 adults at risk of CVD	Randomized controlled trial (FLAVURS study)	Flavonoid-rich fruits and vegetables (berries, citrus fruit, apples, grapes, peppers, onions, broccoli, herbs)	In men, the high-flavonoid fruit and vegetable diet (>15 mg/100 g of total flavonoids) increased endothelium-dependent microvascular reactivity (*p* = 0.017) with +2 portions/d (at 6 weeks) and reduced C-reactive protein (*p* = 0.001) and E-selectin (*p* = 0.0005), while +4 portions/d (at 12 weeks) reduced vascular cell adhesion molecule (*p* = 0.0468). Additionally, +14 portions/d (at 12 weeks) increased plasma NO (*p* = 0.0243) in the group as a whole.	Single-blind design. Limited to low fruit and vegetable consumers. Results may not be generalizable to other populations.
Zong et al. [70]	2024	United States	11,679 adults	Prospective cohort study (NHANES data from 2007–2008, 2009–2010, and 2017–2018)	Total flavonol, isorhamnetin, kaempferol, myricetin, quercetin	Total flavonol intake (~24–288 mg/day, from diet) was associated with reduced CVD-specific mortality (HR: 0.67; 95% CI: 0.47–0.96). Similarly, quercetin intake (~15.07–196.51 mg/day, from diet) and myricetin intake (~1.6–79.76 mg/day, from diet) were linked to reduced CVD-specific mortality risk, with HRs of 0.61 (95% CI: 0.40–0.93) and 0.61 (95% CI: 0.47–0.80), respectively.	Database limitations (lack of dietary flavonol intake data for over half the sample). Potential residual confounding (unadjusted factors like total energy intake and/or supplementation). Study design limits causal inference.
Ponzo et al. [76]	2015	Italy	1658 adults	Prospective cohort study (2001–2003)	Total flavonoids, flavan-3-ols, anthocyanidins, flavanones, flavones, isoflavones	Higher intake of flavonoids (251 mg/day), flavan-3-ols (50.4 mg/day), anthocyanidins (32.9 mg/day), and flavanones (24.2 mg/day) from diet is linked to lower CV risk and all-cause mortality (HR: 0.64, 0.68, 0.66, and 0.59, respectively).	Limited by self-reported dietary data, potential confounding factors, low consumption of certain flavonoid-rich foods.
Xu et al. [69]	2024	United States	8758 adults	Prospective cohort study (NHANES data from 2007–2010)	29 flavonoid compounds and main subgroups (anthocyanidins, flavan-3-ols, flavanones, flavones, flavonols, and isoflavones).	Higher intake of total flavonoids (>169.65 mg/day, from diet) associated with reduced risk of cardiovascular mortality (HR: 0.54, 95% CI: 0.36–0.80). Significant inverse associations for flavonols (>21.30 mg/day, from diet) (HR: 0.41, 95% CI: 0.22–0.78) and flavones (>0.97 mg/day, from diet) (HR: 0.68, 95% CI: 0.29–0.89).	Observational design limits causal inference. Potential misclassification of flavonoid intake due to 24 h recall method. Residual confounding possible. Study population limited to US adults.
Adriouch et al. [75]	2018	France	84,158 adults	Prospective cohort study (2009–2017)	Anthocyanins, catechins, flavonols, dihydrochalcones, proanthocyanidins, dihydroflavonols, hydroxybenzoic acids, stilbenes	For each 10 mg/day intake of anthocyanins (HR: 0.98), catechins (HR: 0.98), and flavonols (HR: 0.94), there was a strong inverse association with cardiovascular disease risk.	Shorter follow-up period, reliance on self-reported dietary data, potential for residual confounding.
**Review Articles**							
**Author**	**Publication Date**	**Study Design**	**Flavonoid(s) Studied**		**Beneficial Effects Observed to Prevent CVDs**		**Limitations**
Liu et al. [85]	2024	Literature review	Various flavonoids (such as quercetin, myricetin, catechin, anthocyanins, nobiletin, hesperidin, formononetin, baicalin, vitexin, silymarin)		Antioxidant, anti-inflammatory, and therapeutic effects through modulation of TLR4-NF-ĸB, PI3K-AKT, and Nrf2/HO-1 pathways. Sources include dietary flavonoids from green tea, soybeans, citrus fruits, red wine, purple grapes, etc.; specific doses are not consistently reported across human, *in vitro*, and animal studies.		Bioavailability and efficacy of flavonoids are not fully addressed due to variability in study designs and dosing.
Yan et al. [90]	2023	Literature review	Herbal monomers baicalin, quercetin, luteolin, naringin		Flavonoid compounds can prevent OS and reduce oxidative damage in cardiovascular diseases by scavenging ROS and enhancing endogenous antioxidant capacity. Sources include red wine, tea, fruits, and citrus fruits, and dietary supplementation. Specific doses are not reported. Effects observed across various studies including human, *in vitro*, and animal research.		Variability in bioavailability and efficacy of different flavonoids not fully addressed.
Yang et al. [91]	2022	Literature review	Citrus flavanones (naringin and naringenin)		Naringenin and naringin (450 mg/day, daily oral intake, in humans) can enhance lipid metabolism and cardiovascular health in *in vitro*, animal, and human studies. Effects were also observed from dietary sources and supplements.		Results across clinical trials are inconsistent. Further research is needed to determine optimal doses, intervention periods, and timing. High *in vitro* doses (e.g., 100 µM) may not directly translate to *in vivo* relevance.
Ciumărnean et al. [83]	2020	Literature review	Various flavonoids (such as: myricetin, quercetin, methyl-flavonol, kaempferol, naringenin, apigenin-7-O-neohesperidoside, tannins, luteolin, rutin, hesperidin, naringenin, pinocembrin, resveratrol, apigenin, cyanidins, phenolic acids, tallianine, diosmetin, catechin, epicatechin, epiafzelechin, gallocatechin)		Multiple cardiovascular benefits in *in vivo* and *in vitro* studies: antiplatelet, anti-inflammatory, antioxidant, antiatherogenic, antihypertensive. Doses were not specified.		Mechanisms not fully understood. Variability in bioavailability and efficacy of different flavonoids.
Fernandes et al. [84]	2017	Literature review	Wine flavonoids (flavanols and anthocyanins)		Wine flavonoids protect CV health by improving endothelial function, reducing LDL oxidation, lowering blood pressure, inhibiting platelet aggregation, and exerting anti-inflammatory and antioxidant effects. Effects shown in *in vivo* and *in vitro* studies. Dietary intake and supplementation, doses were not specified.		Insufficient epidemiological and *in vivo* evidence. Complex variables such as human age, metabolism, and complex wine chemistry complicate the assessment of bioavailability and health-promoting effects.
Maaliki et al. [86]	2019	Literature review	Main subgroups: flavanones, flavan-3-ols, flavonols, flavones, anthocyanins, and isoflavones		Flavanols from apples, pears, tea, grapes, and particularly from cocoa show blood pressure-lowering effects, reduce OS (notably in green tea), improve endothelial function, increase eNOS activity, and induce vasodilation by modulating ion channels. Anthocyanins from vegetables and herbs also lower blood pressure. Flavanones from citrus foods exhibit antioxidant properties and protect endothelial function. These effects are observed in both animal and human studies, though specific doses are not specified.		Variability in bioavailability and efficacy of different flavonoids. Need for more human RCTs to confirm findings.
Grosso et al. [77]	2017	Systematic review and dose-response meta-analysis (22 prospective cohort studies)	Total flavonoids, flavonols, flavones, flavanones, anthocyanidins, proanthocyanidins, lignans		A 100 mg/day increment in total flavonoid intake from diet associated with 4% decreased risk of CVD mortality (P-linearity < 0.001). Dietary flavonoids are associated with decreased risk of CVD mortality.		Limited evidence on specific flavonoid classes and lignans. Potential for residual confounding. Variability in dietary assessment methods across studies.
Wang Xia et al. [95]	2014	Systematic review and meta-analysis (14 prospective cohort studies)	Anthocyanidins, proanthocyanidins, flavones, flavanones, flavan-3-ols, flavonols		An increase of 10 mg/day in dietary flavonol intake is associated with a 5% decreased risk of CVD (RR: 0.95; 95% CI: 0.91–0.99). Diversified intake of flavonoid subclasses through diet is inversely associated with CVD risk.		Potential for residual confounding. Variability in dietary assessment methods across studies.
Rees et al. [88]	2018	Literature review	Various flavonoids (such as: flavanols, epicatechin, procyanidins, quercetin, naringenin, hesperidin)		Flavanol-rich cocoa and green tea exhibit cardioprotective effects, improving FMD response and reducing blood pressure, especially in hypertensive individuals. Blood pressure reductions were noted with cocoa flavanol intake between 450 mg/day and 900 mg/day over one week to one month.		Conflicting results from studies. Need for further research to understand flavonoid effects.
Micek et al. [81]	2021	Dose–response meta-analysis (39 prospective cohort studies)	Total flavonoids, anthocyanins, flavan-3-ols, flavonols, flavones, flavanones, catechins, quercetin, kaempferol		Higher intake of total flavonoids (500 mg/day) is linked to lower CVD risk. Specific doses like 400 mg/day are associated with reduced risks of CHD and stroke. Anthocyanins and flavan-3-ols lower CVD risk; flavonols and flavones reduce CHD risk; flavanones lower stroke risk. Catechins benefit all cardiovascular outcomes. Quercetin and kaempferol are linked to lower CHD and CVD risk, respectively. Doses of other effects are not specified.		Potential for residual confounding. Variability in dietary assessment methods across studies.
Kim and Je [79]	2017	Meta-analysis (15 prospective cohort studies)	Various flavonoid subclasses (excluding flavonols and isoflavones)		High flavonoid intake from diet, specifically around 167.5 mg/day, is associated with a reduced risk of CVD mortality, with a pooled relative risk (RR: 0.86; 95% CI: 0.75, 0.98).		Potential for residual confounding. Variability in dietary assessment methods across studies.
Wang et al. [66]	2014	Meta-analysis of prospective cohort studies (8 studies)	Flavonol		An increase in flavonol intake of 20 mg/day from diet associated with a 14% decrease in stroke risk (summary RR: 0.86, 95% CI: 0.75–0.99).		Meta-analysis, not primary research. Potential for residual confounding. Variability in dietary assessment methods across studies. Inconsistent findings between men and women.
Liu et al. [80]	2017	Systematic review and meta-analysis (10 cohort studies)	Total flavonoids		Marginally significant association between total flavonoid intake (200 mg/day, from diet) and reduced risk of CVD mortality (summary RR: 0.85, 95% CI: 0.70–1.03) and CHD risk (summary RR: 0.74; 95% CI: 0.54–1.02; *p* = 0.069).		Potential for residual confounding. Variability in dietary assessment methods across studies. Marginal significance in results. Significant heterogeneity across studies. Dietary flavonoid intake collected via FFQs (possible recall and measurement bias).
Raman et al. [82]	2019	Systematic review and meta-analysis (157 randomized trials and 15 prospective cohort studies)	Flavan-3-ols		Higher habitual flavan-3-ol intake (e.g., >800 mg/day, from foods and beverages rich in flavan-3-ols) associated with a 13% reduction in CVD mortality and 19% reduction in CHD incidence. Significant improvements in vascular function, blood pressure regulation. Lipid profile improvement and glucose and insulin metabolism, aiding in cardiovascular health.		Considerable heterogeneity in meta-analysis. Many RCTs of poor quality. Reliance on self-reported intake in cohort studies. Need for integrated intake/biomarker approach in future research.
Zhou et al. [92]	2022	Literature review	Various flavonoids (such as: anthocyanins, citrus flavonoids, quercetin, kaempferol, luteolin, hesperidin)		Anthocyanins (320 mg/day, from an anthocyanin mixture) reduce inflammatory response in hypercholesterolemic patients. Citrus flavonoids (270 mg/day) improve cardiovascular parameters, including reducing cholesterol and LDL. Quercetin (500 mg/day) attenuates atherosclerosis. Kaempferol (50 or 100 mg/kg/day, in mice) decreases atherosclerotic lesion area and improves vasorelaxation. Luteolin (50–100 mg/kg, in rats) protects against cardiotoxicity and improves cardiac function. Hesperidin (500 mg/day in humans; 20–40 mg/kg in rats) improves endothelial function and reduces blood pressure.		Bioavailability and dose-responsive effects remain limitations. More studies needed to confirm findings.
Vazhappilly et al. [89]	2019	Literature review	Plant flavonoids (such as: quercetin, pycnogenol, kaempferol, galangin, nobiletin)		Flavonoids reduce the risk of atherosclerosis and atherothrombotic disease by inhibiting excessive tissue factor availability in the endothelium. They also mitigate endothelial dysfunction, reduce oxidative stress, and inhibit platelet aggregation, which are beneficial for cardiovascular health. Doses were not specified, with studies conducted on humans, rats, and *in vitro*.		Dose-responsive effects and bioavailability of flavonoids remain limitations. More studies needed to prove effectiveness as antithrombotic agents.
Chen and Zhang [93]	2021	Narrative review	Various flavonoids (rutin, quercetin, catechin, hesperidin, genistein, apigenin)		Hesperidin improves endothelial function and reduces blood pressure (10–50 mg/kg of glucosyl hesperidin in animals; 500 mg/day for 6 days of hesperidin capsules in humans). Rutin and quercetin regulate and restore elevated blood pressure, promote antioxidant defense, and reduce lipid peroxidation in rats. Catechin-rich green tea decreases systolic and diastolic blood pressure and LDL cholesterol in humans. Doses and sources for rutin, quercetin, and catechin were not specified.		More studies needed to confirm findings.
Jiang et al. [78]	2015	Meta-analysis (15 prospective cohort studies)	Various flavonoids (such as: quercetin, kaempferol, myricetin, nobiletin, hesperidin, sylmarin)		Highest flavonoid intake from diet (doses were not specified) associated with reduced risk of CHD (summary RR: 0.85, 95% CI: 0.79–0.91). Flavonoids improve endothelial function, reduce cardiomyocyte death, minimize postischemic infarct size, and lower inflammation, benefiting cardiovascular health.		Potential for residual confounding. Variability in dietary assessment methods across studies.

NHANES: National Health and Nutrition Examination Survey; HTN: Hypertension; NHS: Nurses’ Health Study; HPFS; Health Professionals Follow-up Study; NIH-AARP: Institute of Health–American Association of Retired Persons; CVDs: Cardiovascular diseases; IHD: Ischemic heart disease; CeVD: Cerebrovascular disease; CV: Cardiovascular; PAD: Peripheral artery disease; FFQ: Food frequency questionnaire; REGARDS: Reasons for Geographic and Racial Differences in Stroke; RCTs: Randomized controlled trials; FMD: Flow-mediated dilation; CHD: Coronary heart disease; OS: Oxidative stress; ROS: Reactive oxygen species.
